# Teaching Strategies During the COVID-19 Pandemic: Tailoring Virtual Learning for Public Health and Cancer Health Disparities Education

**DOI:** 10.3389/fpubh.2022.845400

**Published:** 2022-04-29

**Authors:** Ernesto A. Moralez, Rachel L. Boren, Deanna L. Lebel, Marilyn Drennan, Destiny R. Olvera, Beti Thompson

**Affiliations:** ^1^Public Health Program, St. Lawrence University, Canton, NY, United States; ^2^Evaluation and Effectiveness, New Mexico State University, Las Cruces, NM, United States; ^3^Fred Hutchinson Cancer Research Center, Seattle, WA, United States; ^4^Department of Psychology, New Mexico State University, Las Cruces, NM, United States

**Keywords:** virtual learning, public health, health disparities, barriers to learning, COVID-19, cancer research, virtual workshop

## Abstract

The COVID-19 pandemic has dramatically impacted higher education institutions in the United States (US). Given the dangers of close social interaction in spreading COVID-19, colleges and universities closed their campuses to minimize and often restrict face-to-face instruction of any kind, including supplemental skill development training and experiential learning. In exchange, higher education institutions implemented online learning strategies to continue education for students, including in-person experiential field experiences. This paper describes the adaptation of an in-person experiential field experience into an eight-day virtual workshop as a result of COVID-19 restrictions along with results from participant surveys evaluating pre-and post-test changes in knowledge and their overall assessment of the virtual workshop. This workshop, the Public Health and Cancer Research Workshop (PHCRW), was tailored for students from health-related graduate programs with the primary goal of introducing students to the causes and impacts of cancer disparities in the US/Mexico border region and research related to mitigating those disparities. The course facilitators added a professional development curriculum necessary for student success and the pursuit of advanced degrees such as academic/job interviewing skills and scientific and grant writing. The objectives were for students to (1) understand introductory and intermediate curriculum on public health, cancer, and cancer research; (2) examine the interrelationships among factors impacting public health problems; (3) describe the components of the research process; (4) describe various components of scientific writing; and (5) demonstrate professional strategies associated with school admission and employment. Students completed pre-and post- self-assessments that indicated gains in knowledge about cancer topics, particularly cancer prevention strategies (*M*_pre_ = 3.43; *M*_post_ = 4.43), social determinants associated with cancer (*M*_pre_ = 3.29; *M*_post_ = 4.43), and cancer rates by characteristics (*M*_pre_ = 3.43; *M*_post_ = 4.43). Additionally, students overwhelmingly stated that they appreciated the opportunity to supplement their educational experience in a virtual format. Though the virtual format proved challenging in some respects, students expressed high satisfaction with the workshop. In addition to achieving the goals, the workshop successfully increased students' skills, knowledge, and self-confidence. Despite the last-minute adaptation of the PHCRW, students' satisfaction indicated that this program was an overall success.

## Introduction

The COVID-19 pandemic has dramatically impacted higher education institutions in the United States (US). Given the dangers of close social interaction in spreading COVID-19, colleges and universities closed their campuses to minimize and often restrict face-to-face instruction of any kind, including supplemental skill development training, experiential learning, and field projects. In exchange, higher education institutions implemented virtual learning strategies to continue education for students. This paper describes such an effort to rapidly adapt of an in-person experiential field experience to an eight-day virtual workshop as a response to the changes to existing educational practices because of the COVID-19 pandemic. We wanted to explore if we could effectively present public health curriculum and professional development skills training remotely and address the known barriers associated with virtual learning.

Virtual learning involves using technology and electronic communication to disseminate educational topics (1). Instructors commonly use computers and e-learning strategies to present information, evaluate competencies, and interact with students. There are substantial differences between in-person learning and e-learning. Typically, little interaction occurs, and students have limited engagement with the instructor; further, the pace of learning is different as lectures are presented in their entirety with little time for questioning. On the other hand, e-learning appears to save students time as the pace is accelerated (2). Given the COVID-19 pandemic social distancing protocols, virtual learning has now become the norm for many colleges and universities. Virtual learning, which is 100 percent electronic learning, is qualitatively different from e-learning which has been mainly used as computerized addition to established curricula that gave educational information in various contexts before the COVID-19 pandemic (3). The integration of e-learning into existing curricula has been extensively studied (1, 3), mainly compared to face-to-face learning (4); however, much less research exists on virtual learning.

Research has been published examining the use of virtual learning and e-learning since the pandemic began. These articles have noted the reaction to the push toward e-learning. Al Azzam and colleagues note that many institutions were caught off-guard by the pandemic and the need to close educational institutions and note that students suffered decreased engagement, additional stress, and decreased learning (2). They go on to note that instructors were ill-equipped to deal with the vagaries of the technology, and partially as a result, 67% of students reported they preferred in-person over e-learning. In a systematic review of online strategies, Jnr and Selwyn describe e-learning as a paradigm shift in learning that forced changes in instruction (5). Although they argue that virtual learning has advantages (e.g., flexibility in preparing courses), they note that weaknesses include poor assessment of students' skills and development. Further, others have stated that virtual learning fails to provide the ideal teaching and learning environment especially when instructors lack the training in the technology (6). A call for innovative approaches in virtual learning to stimulate the experience also has been made (7). To date, virtual learning does not appear to appeal to students with more than half expressing an unfavorable position on virtual learning and almost 74% stated they are unsatisfied with the teaching in virtual learning (8). Thus, our work focused on innovative instruction and much faculty interaction.

Regmi and Jones (1) conducted a systematic review of factors related to health sciences education before the current pandemic. In the summary of 24 studies, several facilitators and challenges to e-learning were identified and explored. Although not unique to the COVID-19 situation, those factors may be relevant for virtual learning in today's environment. From the systematic review, facilitators foster understanding because students can review online information at their own pace. A second facilitator was learning in practice; students could see the implementation of skills in practice. A third facilitator was a systematic approach to learning; students could move from simple to complex ideas by integrating theory into practice. Finally, the authors noted that the integration of e-learning into curricula fosters independent and interactive learning. For the most part, those preceding facilitators may also pertain to the current virtual learning environment necessitated by the pandemic. However, e-learning as a support for learning environments, as explored by Regmi and Jones (1) and others, might be substantially different from a mixed and integrated approach. For example, virtual learning in the context of COVID-19 may not foster learning at one's own pace in the context of synchronous lessons. Similarly, it may be difficult to see the implementation of skills in a virtual environment (3). Nevertheless, aspects of the cited facilitators to e-learning do apply to the current virtual learning situation.

Challenges to virtual learning are plentiful. An immediate problem is the lack of technical skills to present curricula and use online equipment to respond to the curricula (8, 9). Both students and faculty may have limited technology skills; this might mean very labor-intensive work and the inability to make class time interactive and engaging. For students, this may indicate a failure to be interactive and ask faculty questions without having appropriate Information Technology (IT) skills. Thus, a significant learning curve is necessary to interact effectively online. Another challenge with virtual learning is that everyone is required to have access to a reliable internet connection or access to technology that would allow them to participate in this program. In the planning process, we attempted to address as many of the known barriers to virtual learning. We familiarized ourselves with the virtual learning platform and piloted the workshop before the start date to practice screen sharing, using the breakout room feature, and having multiple hosts for each session. Additionally, we hired a student whose primary responsibility was to monitor all technical aspects of the workshop to reduce any anticipated difficulties with remote learning.

Another challenge is student dissatisfaction with online learning; this may include students feeling isolated and lacking engagement. Some students report feeling anxiety and stress. Particularly noted among graduate students, this anxiety and stress are due to a feeling of isolation and lack of community in the training process (10). Others have noted anxiety and stress due to the pandemic (2). Others note that online learning affects self-discipline and students report feeling less motivated to achieve mastery in an online setting (1). To help keep students engaged, this workshop also was dynamic in offering instruction-based learning, guest presentations, and organized discussions for students. Students also complain of “computer screen fatigue” from spending long hours online (11, 12). In an attempt to mitigate this feeling of online fatigue, we incorporated multiple breaks into our workshop schedule. It has also been noted that some disciplines are less amenable to virtual learning than others; for example, public health, especially health disparities research, requires guided reflection and discussion to promote a solid understanding of different cultures, inequities, and effects of social determinants of health (13, 14). Interaction between faculty and student with channeled questioning and thinking reflecting the pedagogy that one's learning is never done (15) is a common initiator of public health curricula.

## Pedagogical Framework

Student-learning programs have been offered to acquaint underrepresented students with public health and cancer disparities as a significant part of an NCI-funded partnership grant between New Mexico State University (NMSU) and the Fred Hutchinson Cancer Research Center (Fred Hutch). In the past, these programs were field-based experiences that allowed students to experience first-hand the barriers faced by people who suffered from health disparities and inequities in the US-Mexico border region. Switching to a virtual curriculum eliminated much of the experiential learning; however, we needed to develop a curriculum that maintained the understanding of health equity and social determinants. Thus, we leveraged the facilitators and barriers to e-learning outlined in the systemic review to develop a virtual workshop to simulate the questioning, discussion, and interaction with colleagues and faculty that we presented in our previous curriculum. We followed the thinking and writing of Freire, who recommends learning through ongoing questioning (16).

The integration of virtual learning into existing curricula has ranged from semester-long periods to shorter periods, but few experiences lasted only 2 weeks. We report on a 2-week graduate workshop, conducted entirely online, on public health and cancer health disparities. Because the focus was on public health and health disparities, the workshop fell into the category of being challenging to present online (14). Despite that challenge, we aimed to reduce the barriers to virtual learning and provide students with a rich and practical learning experience using innovative techniques and strategies. A set of learning goals, identified below, focused not only on the substance of public health and health disparities but also, in the interest of generating competent public health practitioners, we focused on scientific writing and professional employment strategies. In this report, we conduct a mixed-methods evaluation to assess whether the participants attained the goals and objectives of the workshop and the related skills.

## Methods/Environment

### Recruitment

The Partnership for the Advancement of Cancer Research (PACR), the joint NMSU and Fred Hutch collaboration, ordinarily hosts a 9-week summer internship experience for Fred Hutch/University of Washington (UW) and NMSU graduate students at Fred Hutch. Like many other summer programs, this internship, that had accepted students, was canceled because of the COVID-19 pandemic. The students accepted to this summer internship were contacted via email and invited to attend the Public Health and Cancer Research Workshop (PHCRW), a 2-week virtual learning experience. We then asked these students to share the program announcement with their colleagues. To avoid creating an additional burden on students who were already overtaxed by the transition to remote learning, we elected not to have a formal application process; instead, we admitted the first eight graduate students interested in the program. We deliberately limited the number of students invited to participate to ensure all could be actively engaged and receive mentorship and support.

Participants submitted a resume to hold their spot in the program. Four participants were enrolled in master's programs at UW and four in master's degree programs at NMSU and all eight participants expressed an interest in pursuing medical or post-graduate school education (e.g., doctoral degree). Disciplines of participating students included public health, nursing, global health, and medical anthropology. The students were ethnically diverse, with six of the participants being of non-white ethnicity.

### Program Description

The PHCRW provided the students with a multi-faceted virtual experience to cover topics pertinent to understanding health disparities commonly seen in the US-Mexico border region. In addition, the workshop also covered supplemental curriculum critical to student success, including scientific writing, grant/research proposal development, statistical data analysis, and professional development (i.e., interviewing for job/graduate programs, writing cover letters and resumes/CVs). The PHCRW consisted of eight modules (Table 1) with a curriculum and activities designed to help participants meet the following workshop goals:

- Understand introductory and intermediate curriculum on public health, cancer and cancer research, and active projects at the Fred Hutch, UW, and NMSU and professional development for students.
- Examine the interrelationships between factors that impact public health problems (i.e., cancer), including environmental, social, behavioral, and economic factors.
- Describe the components of the research process and designing a study, including how to determine the purpose of a study and develop research questions.
- Describe various components of scientific writing, including abstract and grant writing.
- Demonstrate professional strategies associated with school admission and employment, including developing a CV/resume, negotiating salaries, and interviewing skills.

**Table 1:** Descriptions of the workshop modules.

**Module title**	**Module topics**
Introduction to Public Health Research	•A brief overview of public health •Key public health terms •Determinants of health •Public health research, policy, and practice
Introduction to Cancer	• What is cancer? • Prevention and interventions • Cancer screening modalities
Epidemiology of Cancer	• Disease frequency • Exposures • Rates and distribution • Trends and problems
Scientific Writing	• Basic writing principles • Writing a scientific abstract • Writing specific aims
Health Disparities and Cancer	• Health inequities • Social determinants of health • Mortality and survivor rates • Racial disparities in treatment • “Upstream” approaches • Research areas and opportunities
Quantitative Research	• Overview of study designs • Case-control studies • Cohort studies • Regression Analysis
Introduction to STATA Quantitative Analysis Software	• Categorizing variables • Recoding variables • Creating new variables • Measures of association
Professional Development	• Writing a CV/Resume • Writing a cover letter • Interviewing skills • Negotiating your salary

**Figure 1:**
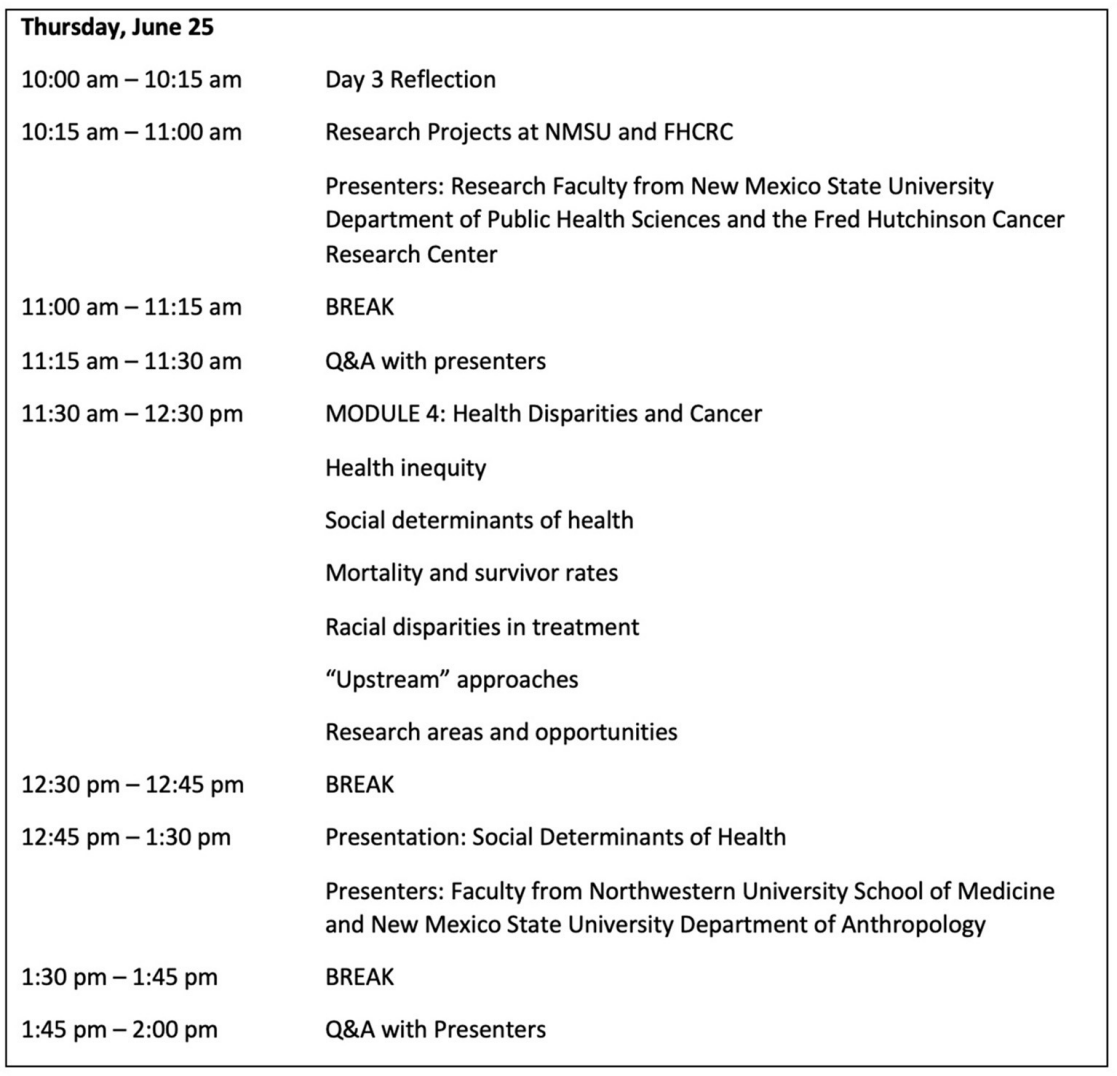
Sample agenda from the Public Health and Cancer Research Workshop (Day 4 of 8)

The PHCRW took place over 8 days, synchronously, with daily schedules lasting approximately 4 h with breaks (Figure 1). The workshop's focus came from student feedback from past iterations and input from research mentors involved with the PACR partnership. PHCRW students met virtually with public health experts to discuss the causes of health inequities and disparities and to learn about ongoing research projects related to social determinants of health and cancer prevention in underserved populations. Additionally, participants met with scientific and grant writing experts and received real-time feedback on their writing, experienced mock interviews, and practiced negotiation procedures.

The PHCRW and associated data collection were approved by the NMSU and Fred Hutch Institutional Review Boards (IRB). Participating students completed an informed consent document approved by the Fred Hutch IRB (file #6617) and the NMSU IRB (file #11709), respectively.

### Data Collection

Students were given a brief survey at the beginning and end of the workshop to assess if and to what extent the workshop goals (listed above) were achieved and to obtain feedback about the entire workshop experience. The students completed the pre-test survey before the workshop began and then completed the post-test survey at the end of the last day.

The questionnaires asked students to rate their knowledge or skill level in critical constructs of interest to determine if there were any changes from pre to post and where these were most pronounced. Students were presented with a scale from One (Learner) to Six (Expert), with descriptors for the level of knowledge or understanding that matched each level. The categories are 1: Learner: I have little to no knowledge about this topic. 2: Beginner—I have a common knowledge or an understanding of basic techniques and concepts but no more than this level. 3: Novice—I have the level of experience gained like a trainee on-the-job. I consistently need help when performing this skill. I would not feel comfortable answering questions, but I have a better understanding of this topic than others not in my field. 4: Intermediate—I can complete tasks in this area requested, though I may need help from an expert from time to time, I can usually perform this skill independently. I know this area pretty well, and if asked, could answer basic questions about this area. ·5: Advanced—I can perform the actions associated with this skill without assistance, but I could improve to become an expert and can answer most, but likely not all, questions. 6: Expert—I know this area extremely well and if asked, could provide guidance and answer questions in this area. These scales were adapted from the National Institutes of Health Competencies Proficiency Scale so individuals could evaluate their capabilities to perform job-related skills.

The last part of the pre/post questionnaires prompted students to rate their confidence level in the final set of core areas within the learning goals. Again, the five-point scale ranged from “Not at All Confident” to “Extremely Confident.”

On the post-survey only, students also were given a set of questions that asked about their experience in the program with questions that asked them to rate their level of agreement with statements about their experience, such as “The module instructors were knowledgeable,” “The module topics built upon one another,” and “The online delivery was effective for helping me learn.” Students were also asked to provide open-ended feedback about their responses, identify the most impactful part of the workshop, and offer suggestions for improving the program for future cohorts. To separate the feedback about the technology and delivery among the open-ended feedback responses, students could openly comment on the effectiveness of the Zoom (a virtual meeting platform) delivery in a separate open-ended question.

To standardize pre-and post-survey data collection, the evaluator joined the first and final Zoom meetings to collect the data from students. The administration of each survey took approximately 20 to 25 minutes each.

### Data Analysis

REDCap (Research Electronic Data Capture) was used to collect and store all data. REDCap is a secure, web-based software platform designed to support data capture for research studies (17, 18).

With the small sample size, data analysis was more exploratory, as inferential statistics would not be appropriate for the n of seven respondents to the post-survey. For the closed-ended responses that asked students to rate their knowledge or confidence at the start and end of the summer and post-only questions about the summer experience, a weighted mean was calculated from the response options for each survey administration. Open-ended responses were summarized by theme(s). The focus for these questions was to identify program components that worked well and those that need to be changed for the better attainment of program goals.

## Results

A total of eight students participated in the workshop. One student did not complete the post questionnaire. Of the seven respondents, one student was in a doctoral program, and the rest were pursuing master's degrees, all in public and population health areas. Five students were females, two were males, and one participant indicated they were a first-generation college student. Regarding race/ethnicity, two students were of more than one race, Black or African American, White or Hispanic, and one student was American Indian or Alaska Native.

After examining the pre-post changes in self-assessments of knowledge and self-confidence in critical areas, there were topics where students had more substantial improvements than in others. No questions indicated that student self-assessments decreased from pre to post or stayed the same. Table 2 shows that students increased their knowledge of quantitative methods and understood confidence intervals, correlations, regressions, and data management. Also shown in Table 3, students showed most gain in assessments of their knowledge in cancer topics, particularly cancer prevention strategies (*M*_pre_ = 3.43; *M*_post_ = 4.43), social determinants associated with cancer (*M*_pre_ = 3.29; *M*_post_ = 4.43), and cancer rates by characteristics (*M*_pre_ = 3.43; *M*_post_ = 4.43). They also exhibited a notable increase in their knowledge about data management techniques (*M*_pre_ = 3.57; *M*_post_ = 4.29). Student confidence in different research skills also increased. However, these increases were not as pronounced as the areas above.

**Table 2:** Mean skill levels of participants pre-and post-workshop.

Skills*	**Pre-workshop mean skill level**	**Post-workshop mean skill level**
Identifying quantitative research designs	4.57	4.71
Interpreting confidence intervals	4.57	4.86
Interpreting a correlation coefficient	4.14	4.57
Interpreting regression coefficients	3.86	4.29
Data management techniques	3.57	4.29

*Scores range from 1: Learner to 6: Expert.

**Table 3:** Mean level of knowledge of participants pre-and post-workshop.

Knowledge level*	**Pre-workshop mean level**	**Post-workshop mean level**
Cancer prevention strategies	3.43	4.43
Cancer screening modalities	3.43	4.29
Social determinants associated with cancer	3.29	4.43
Cancer rates by Characteristics	3.29	4.43

*Scores range from 1: Learner to 6: Expert.

In terms of skills, participants were asked a series of questions about their ability to: identify quantitative research designs, interpret confidence intervals, interpret a correlation coefficient, interpret regression coefficients, and understand data management techniques. We asked participants to give examples of the following and then to rate their level of expertise with the topic. The categories included, “give an example of”: cancer prevention strategies, cancer screening modalities, social determinants associated with cancer, and cancer rates by characteristics.

As mentioned, the workshop included items such as writing a literature review, grant, specific aim, as well as ability to collaborate with students, interview for graduate school, and negotiate a salary. Table 4 shows the increases in confidence to perform those tasks. As can be seen, there were great advances in approaching faculty, in various writing tasks, and in negotiating a salary. Less confidence was displayed in collaboration with other students, in making presentations, and in interviewing for graduate school.

**Table 4:** Mean confidence of participants to perform skills pre-and post-workshop.

**Confidence**	**Pre-workshop Mean Level**	**Post-workshop Mean Level**
Conducting literature Review	3.86	4.14
Presenting in a professional setting	3.29	3.86
Collaborating with other students	4.14	4.43
Approaching faculty for assistance/input	3.57	4.14
Writing a grant application	2.57	3.71
Writing a specific aim	3.14	4.14
Interviewing for graduate school	3.29	3.86
Using strategies to negotiate my salary at my next job	1.57	3.43

*Scores range from 1 (not at all confident) to 5 (extremely confident).

Post-only feedback about the experience was overall very positive. Students uniformly strongly agreed that the instructors were knowledgeable, the modules were organized, the program staff was helpful if students needed them, and that overall, this workshop was a valuable experience (Table 5). None of the statements had any disagreement responses.

**Table 5:** Mean post-workshop rating of the overall experience of the learning experience.

Item*	**Mean rating**
The instructors were engaging	3.86
The instructors were knowledgeable	4.00
The content was clearly presented	3.86
The topics built on each other	3.71
The content was organized	4.00
The online delivery was effective at keeping me engaged	3.57
The online delivery was effective for helping me learn	3.43
The program staff were helpful if I needed them	4.00
Overall, this workshop was a valuable experience	4.00

*Rating varies from 1 (strongly agree) to 4 (strongly disagree).

Students cited a few primary areas they considered the most impactful part of the workshop when completing the open-ended questions. These included professional skills (*n* = 3), the sense of community (*n* = 2), and meeting new people/learning about new career paths (*n* = 2). Students liked the people they met in the workshop. As one student noted,

*[I liked] meeting so many people who are passionate about what they do. Having the ability (in a small group) to ask questions and get involved in robust discussions with facilitators and lecturers*.

Another student commented,

*Honestly, just the small learning community. In my graduate program, there aren't many opportunities to learn with a small group of students and feel comfortable asking questions*.

Students who talked about professional skills described how they learned about critical areas such as writing, resume construction, and networking. One student said: [I liked] “*the scientific writing modules. The introduction to the diverse set of public health projects and the networking.”*

When asked about what the students valued most about the workshop (Table 5), many of the students highlighted the interactions with the PHCRW faculty learning about their work and their paths to their current role:

*Meeting so many people who are passionate about what they do. Having the ability (in a small group) to ask questions and get involved in robust discussions with facilitators and lecturers*.

*The most impactful part was learning about the different paths each public health educator took. I often wonder about my own path and learning how they all got there as well as the lessons learned was so reassuring in what I want to do*.

*Meeting faculty and hearing their real thoughts*.

Student responses also fit into three main areas when asked about improvements they suggested for the workshop. Most often noted improvements or changes were focused on how the quantitative content was presented (*n* = 3). Students shared this went very fast or that they would have preferred a different statistical software. As one student noted:

*The only day that I thought could have been better was our quantitative analysis. I think it's an important component but was taught too fast. Fortunately, I was able to keep up because I have experience with Stata, but I don't think it was as useful for many other people. The way that she was teaching the content was more for people that already knew about Stata*.

Another concrete suggestion for workshop improvements involved modifying the layout of the day to allow for more breaks or scheduling more time with other faculty (*n* = 2), with one student specifying,

*I think more time for the workshop would be better...as a time for eating. I felt like I could not eat in between the 10-to-15-minute breaks, and a short lunch break of 30 minutes would help with that and limit the Zoom exhaustion*.

Finally, when asked specifically about the online delivery and what can be improved to be more effective as an online program, most students (*n* = 4) offered specific suggestions about the scheduling, such as putting in more breaks or more times for networking with each other, “*Break things up with a variety of different activities (add videos, break-out rooms, etc.). It really helps with Zoom fatigue!*”

The other responses to this question indicated no improvements were needed specific to the online delivery with one participant responding,

“*the online delivery great! I loved being able to interact so easily and to have the one-on-one interaction as well as group work which made the experience more face-to-face like.”*

## Discussion

Although the COVID-19 pandemic forced many supplemental opportunities (e.g., internships, study abroad programs) for students in higher education to be canceled, we were able to modify the PHCRW to provide students with a meaningful summer experience. Students overwhelmingly appreciated the opportunity to supplement their educational experience, even in a virtual format. The facilitators of the PHCRW covered many of the critical topics of public health and cancer health disparities, in addition to skills necessary to student success and pursuit of advanced degrees. Knowing the existing barriers to virtual learning discussed in previous literature, we were intentional with our scheduling, offering several breaks and keeping sessions short to reduce “Zoom fatigue” (12) and switching between instruction-based learning, guest presentations, and organized discussions. Even with the known difficulty of this online format, students expressed high degrees of satisfaction with the workshop both quantitatively and qualitatively. Specifically, the workshop successfully increased students' abilities in three areas: skills, knowledge, and self-confidence. Despite focusing on topics that are thought to be challenging to teach in an electronic learning situation, our students uniformly increased in all of these areas. Overall, students had few suggestions about how the program could be improved. The only suggestion for enhancement of the program was the need for more frequent breaks.

The PHCRW facilitators intentionally developed the workshop to address the challenges to virtual learning (1). To accommodate technical issues, we hired a student worker trained to manage the online platforms and handle any problems experienced by the students and presenters. The workshop schedule included time for students to interact with one another, share ideas and reflections, and schedule break-out sessions for discussions about the topics; this helped address isolation and fostered engagement. We also had several breaks throughout the day and kept daily sessions under a total of 5 h. Finally, to address the challenges of teaching public health and health disparities in a virtual format, presenters were encouraged to describe the communities that they worked with, including cultural practices, specific reasons for the differences experienced in those communities, and the current projects mitigating adverse health outcomes. All presentations included allocated time for questions. Many presenters shared their own lived experiences (e.g., what they studied, how they got involved in their current work), which was noted by the students as one of the more impactful workshop components.

Strengths of the PHCRW included providing the opportunity for students to engage in real-time discussions with research faculty. Further, the virtual program allowed nationally known leaders in critical areas such as science writing, curriculum vitae creation, and negotiation skills from other universities and research centers to disseminate their knowledge to the students; this component would have been nearly impossible in a traditional face-to-face format. Another strength of the workshop was the involvement of several black, indigenous, and other people of color [BIPOC] faculty (including the lead facilitator, which was mentioned by one of the students as was most impactful about the workshop:

…*having a POC [person of color] lead this experience was really powerful! I've actually not had a single Latinx professor in my entire graduate study experience, so it was so great to be able to learn in this environment*.

Despite the challenging environment caused by the pandemic, virtual learning has shifted the way we will do education moving forward; that is, we will build on the strengths of this program. In this collaborative grant between NMSU and Fred Hutch, this experience has changed how future meetings will be held between the partnering institutions. As a result of the PHCRW, we will encourage collaboration among students from different institutions and invite researchers and other key experts to present in classrooms using virtual platforms.

## Limitations

The experience was not without limitations. The small sample size makes it difficult to extrapolate or generalize results to a larger sample. However, it is important to note that the PHCRW was intentionally designed to limit the number of students to overcome some of the challenges of virtual learning; thus, a small sample size was necessary. The surveys used were not tested and only distributed once so we could not assess any behavioral outcomes, however, the workshop assessment questions were developed by an experienced program evaluator and specifically designed to measure changes in skills and knowledge. Nevertheless, the pre-and post-survey data also show that the workshop activities and the format were well-received by the students and can be used to develop future virtual public health and health disparity-focused workshops.

## Conclusion

In this work, we describe a cancer health disparities workshop for graduate students from health-related disciplines converted to a virtual format due to university COVID-19 restrictions. The results from this workshop evaluation indicate that virtual learning could be successful in teaching a sample of graduate students about public health and cancer health disparities. Students who completed the workshop significantly increased their knowledge related to public health and cancer health disparities and showed improvement in confidence related to other professional skills (e.g., scientific writing, CV preparation) critical to success in academic careers. Our findings indicate that virtual learning can be an effective platform to teach knowledge critical to public health education. It also demonstrates that addressing the barriers established in recent literature is crucial to careful planning and ensuring that students have a meaningful and valuable experience. Qualitative responses noted that the workshop was well-received and that the students felt engaged and that they appreciated being taught by people of color. The students' positive responses to the PHCRW format and scheduling including inviting guest speakers from other institutions, keeping daily agendas short with time for breaks, and encouraging constant dialogue can help design future public health curriculum workshops. Additionally, future public health-based workshops should incorporate virtual learning as part of the ongoing collaboration between the partnering institutions.

## Data Availability Statement

The raw data supporting the conclusions of this article will be made available by the authors, without undue reservation.

## Ethics Statement

The studies involving human participants were reviewed and approved by Fred Hutchinson Cancer Research Center, New Mexico State University IRB. The patients/participants provided their written informed consent to participate in this study.

## Author Contributions

EM, BT, RB, and DO: the conceptualization of the manuscript consisted of the following contributors. EM, BT, RB, DO, and MD: a program evaluator was added to the project and helped with the methodology along with the primary authors. EM, BT, RB, MD, and DL: a student was added to help with writing and editing. DL: handled the manuscript submission process. All authors contributed to the article and approved the submitted version.

## Conflict of Interest

The authors declare that the research was conducted in the absence of any commercial or financial relationships that could be construed as a potential conflict of interest.

## Publisher's Note

All claims expressed in this article are solely those of the authors and do not necessarily represent those of their affiliated organizations, or those of the publisher, the editors and the reviewers. Any product that may be evaluated in this article, or claim that may be made by its manufacturer, is not guaranteed or endorsed by the publisher.
